# Data-Driven Identification of Risk Factors of Patient Satisfaction at a Large Urban Academic Medical Center

**DOI:** 10.1371/journal.pone.0156076

**Published:** 2016-05-26

**Authors:** Li Li, Nathan J. Lee, Benjamin S. Glicksberg, Brian D. Radbill, Joel T. Dudley

**Affiliations:** 1 Department of Genetics and Genomic Sciences, Icahn School of Medicine at Mount Sinai, 770 Lexington Ave., 15^th^ floor, New York, NY 10065, United States of America; 2 Department of Medicine, Icahn School of Medicine at Mount Sinai, One Gustave L. Levy Pl, New York, NY 10029, United States of America; 3 Department of Population Health Science and Policy, Icahn School of Medicine at Mount Sinai, One Gustave L. Levy Place, New York, NY 10029, United States of America; Toronto Western Hospital, CANADA

## Abstract

**Background:**

The Hospital Consumer Assessment of Healthcare Providers and Systems (HCAHPS) survey is the first publicly reported nationwide survey to evaluate and compare hospitals. Increasing patient satisfaction is an important goal as it aims to achieve a more effective and efficient healthcare delivery system. In this study, we develop and apply an integrative, data-driven approach to identify clinical risk factors that associate with patient satisfaction outcomes.

**Methods:**

We included 1,771 unique adult patients who completed the HCAHPS survey and were discharged from the inpatient Medicine service from 2010 to 2012. We collected 266 clinical features including patient demographics, lab measurements, medications, disease categories, and procedures. We developed and applied a data-driven approach to identify risk factors that associate with patient satisfaction outcomes.

**Findings:**

We identify 102 significant risk factors associating with 18 surveyed questions. The most significantly recurrent clinical risk factors were: self-evaluation of health, education level, Asian, White, treatment in BMT oncology division, being prescribed a new medication. Patients who were prescribed pregabalin were less satisfied particularly in relation to *communication with nurses* and *pain management*. Explanation of medication usage was associated with *communication with nurses (q = 0*.*001)*; however, explanation of medication side effects was associated with *communication with doctors (q = 0*.*003)*. *Overall hospital rating* was associated with *hospital environment*, *communication with doctors*, and *communication about medicines*. However, patient likelihood to recommend hospital was associated with *hospital environment*, *communication about medicines*, *pain management*, and *communication with nurse*.

**Conclusions:**

Our study identified a number of putatively novel clinical risk factors for patient satisfaction that suggest new opportunities to better understand and manage patient satisfaction. Hospitals can use a data-driven approach to identify clinical risk factors for poor patient satisfaction to support development of specific interventions to improve patients’ experience of care.

## Introduction

Patient satisfaction lies at the core of the Institute of Medicine’s six “Aims for Improvement” and thus, it serves as an important measure of high-quality healthcare delivery in the United States[1]. With the passage of the Affordable Care Act, patient satisfaction has taken center-stage because of the significant impact it has on the Value-Based Purchasing (VBP) program[2]. The Centers for Medicare & Medicaid Services (CMS), along with the Agency for Healthcare Research and Quality (AHRQ), developed the HCAHPS (Hospital Consumer Assessment of Healthcare Providers and Systems) Survey to provide a standardized survey instrument and data collection methodology for measuring patients' satisfaction with their hospital care experience. The HCAHPS survey is the first nationwide, publicly reported survey of its kind used to evaluate and compare hospitals at both state and national levels. Unlike other measures of healthcare quality, HCAHPS scores aim to reflect the extent to which providers and patients reach a common understanding of the patient’s situation[2]. In addition to being recognized as an important outcome unto itself, patient satisfaction has been correlated with higher adherence to treatment, reduced 30-day readmission rates, and decreased inpatient mortality[3]^,^[4]. Therefore, increasing patient satisfaction is an important goal because it aims to characterize a healthcare delivery system that is more effective, more efficient, and patient-centered.

Increased transparency and mandates tied to potential financial penalties have prompted hospitals to focus on improving patient satisfaction. Although studies outline efforts to enhance the interpersonal component of inpatient clinical care, the challenge remains how to implement these practices consistently and effectively across different patient populations [5–9]. Furthermore, many of these interventions employ so-called “best practice” protocols that have face validity but whose efficacy may not have been extensively studied and work under the assumption of an undifferentiated mass of patients[5]. This type of global effort initiative attempts to benefit all patients by treating them as a whole; however, research suggests that hospitals may be able to more meaningfully improve a patient's hospital experience by recognizing and addressing an individual patient's unique needs[10]. Identifying the underlying factors that influence specific dimensions of the patient’s hospital experience will be a crucial step toward developing more targeted interventions for subgroups with the most pronounced needs, thereby achieving better outcomes and more efficient utilization of resources.

Prior studies have demonstrated the effect of both patient and disease-specific factors on the patient experience. For example, a national observational study revealed that doctor and nurse communication domains of the HCAHPS survey varied substantially by patient health status, race/ethnicity, age, and education [10–13]. Another study using the pilot version of the HCAHPS survey demonstrated that patients with different diagnostic categories varied according to how they scored different dimensions of care [14]. Additionally, dimensions of the HCAHPS survey may differ across hospitals. Studies indicate that hospital-level factors, including non-profit status, small size, rural location, and a high nurse-to-patient ratio [15, 16] associate with patient satisfaction outcomes. These findings suggest that targeting patients with the lowest satisfaction requires an understanding not only of patient-specific characteristics, but also of the many disease and hospital-specific factors that contribute to patient experience.

Patient satisfaction is a complex phenomenon influenced by factors beyond what standardized surveys capture. To fully understand what drives the variations in how patients respond to each of the core questions of the HCAHPS survey there is need for more expansive research on clinical aspects of care pertaining to variables such as disease status, care history, administered treatments and procedures, and characteristics of the clinical staff and hospital environment. A limitation of previous studies on patient satisfaction is that they tend to be hypothesis-driven, and, therefore, tend not to consider all available features and data that might inform on patient satisfaction outcomes. A data-driven approach would evaluate other potentially informative data, such as broad clinical variables associated with the patient visit, to explain the variability.

In the current study, we develop and apply an integrative, data-driven approach to identify clinical risk factors that associate with patient satisfaction outcomes. We leverage data extracted from electronic medical records (EMR) for an inpatient cohort from 2010 to 2012 and develop a risk factor profile for each individual question of the HCAHPS survey at our large academic medical center. To our knowledge, this study represents the first to evaluate risks of a poor inpatient experience using integrated clinical features from various resources including high-dimensional clinical data obtained from the EMR.

We identified 102 unique risk factors associating with 18 questions in the HCAHPS survey. Of these 102 risk factors, 38 risk factors consistently presented more than once. We constructed a "questions network" to assess the similarities among questions based on their shared clinical risk factors. The results identify specific domains and aspects that may be used to improve patient satisfaction and possibly other outcomes by targeting areas and prioritizing certain features for at-risk subgroups of the inpatient population. In doing so, we outline a methodology that can be utilized by other healthcare systems to emulate our approach.

## Methods

### Data sources

This study reports an observational study on a single center experience at the Mount Sinai Hospital (MSH) in New York City. We enrolled adult patients who were admitted through the emergency department, hospitalized, and then discharged from the Medicine Service at Mount Sinai between January 2010 and December 2012. We randomly mailed adult patients between 48 hours and 6 weeks after discharge. A total number of 1,771 unique patients who completed the HCAHPS patient satisfaction survey were included in this analysis. Patient characteristics and clinical features were collected from InfoAssist (a billing and cost database at MSH) and United Healthcare (UHC, http://www.uhc.edu). The study was specifically governed and approved by Institutional Review Board approval at MSH (IF1700346 GCO 13–1583). This retrospective study could not have been reasonably carried out if all records accessed required informed consent. Our IRB reviewed the study proposal and granted us a waiver of informed consent and a waiver of HIPAA authorization. The data set used in the analysis was extracted and anonymized from our clinical data warehouse in accordance with institutional, state, and federal clinical data privacy policies and standards.

### Survey inclusion criteria and components

The HCAHPS Survey is administered to a random sample of patients continuously throughout the year. The patient inclusion criteria are: 1. ≥ 18 years at the time of admission; 2. At least one overnight stay in the hospital as an inpatient; 3. Non-psychiatric Medicare severity diagnosis related groups (MS-DRG)/principal diagnosis at discharge; 4. Be alive at time of discharge. On average, the survey response rate from patients was 33% (22–45% each year). We received 1,771 completed surveys from 360, 772 and 639 patients in 2010, 2011 and 2012 respectively.

The HCAHPS survey is comprised of 27 questions in total, with 18 individual questions covering multi-dimensional components of care in the hospital and 9 screening and demographic questions used for adjusting the mix of patients across hospitals for analytical purposes (S1 Table). The 18 individual questions (Q1-18) consist of six composite topics including: 1. Communication with nurses (Q1-3, n = 3); 2. Communication with doctors (Q4-6, n = 3); 3. Responsiveness of hospital staff (Q9-10, n = 2); 4. Pain management (Q11-12, n = 2); 5. Communication about medicines (Q13-14, n = 2); 6. Discharge information (Q15-16, n = 2). Additionally, there are two individual-centered topics including: 1. Cleanliness of hospital environment (Q7, n = 1); 2. Quietness of hospital environment (Q8, n = 1). Finally there are two globally focused topics including 1. Overall hospital rating (Q17, n = 1); 2. Willingness to recommend hospital (Q18, n = 1).

We used the ‘top-box’ scoring system to categorize the difference ordinal levels of the responses for each question. ‘Top-box’ is the most positive response to HCAHPS survey items and has been suggested more recently as a better way of reporting satisfaction scores (http://www.hcahpsonline.org) [17]. The ‘top-box’, or most favorable, response is "*Always*” for five HCAHPS composites (Communication with Nurses, Communication with Doctors, Responsiveness of Hospital Staff, Pain Management, and Communication about Medicines) and two individual topics, "*Yes*" for the sixth composite, Discharge Information, "9” *or “10 (best)*" for the Overall Hospital Rating, and *"Would definitely recommend”* for the Willingness to Recommend Hospital. The scores in this study are reported as binary variables using the ‘top-box’ scoring system.

### Clinical data extraction and integration

We compiled comprehensive clinical data (n = 266) through integrating information from various sources. First, we collected 28 clinical features from InfoAssist and UHC, including patient demographics, in-hospital primary diagnosis based on ICD9-CM codes, procedures during their in-hospital stay, admission/discharge information, mortality risk, and insurance carrier information (S3 Table).

Secondly, we collected 7 CMS questions concerning education levels, self-evaluation of health status, Hispanic origin, pain management during the stay, and discharge information from the patient satisfaction survey.

Thirdly, we collected all lab measurements one year prior to the date of discharge to evaluate the potential risks from the Mount Sinai Data Warehouse (MSDW). The MSDW is a de-identified EMR repository consisting of 182 million lab test results and 94 million medications on more than 4 million unique patients cared at Mount Sinai Hospital. We used the reference ranges from MedlinePlus at National Library of Medicine to determine the lab value. Values outside the reference range were defined as being “abnormal low” or “abnormal high”, depending on direction. For a given lab test, we compared the maximum and minimum lab values to the reference range if multiple tests had been performed on a patient during the one-year analysis time frame. Values below than low end of the reference range were defined as “abnormal low”, and those greater than the high limit of the reference range were “abnormal high”. Accordingly, patients were defined as “normal” if their lab results were within reference ranges. We included lab tests with no more than 10% missing values from our patient cohorts, yielding a total of 28 lab measurements in the analysis. Next, we collected the 200 most prescribed medications one year prior to the date of discharge.

Finally, we categorized diagnoses and procedures into unique groups based 542 unique ICD-9-CM codes in our cohort using the Clinical Classifications Software (CCS). The CCS is a tool developed at AHRQ for clustering patient diagnoses and procedures into a manageable number of clinically meaningful categories[18] at different levels. The single level of CCS is used to classify all diagnoses and procedures into unique, one-dimensional groups based on ICD-9-CM codes. The multi-level characterization of CCS is used to group single-level CCS categories into broader body systems or condition categories (e.g., "Diseases of the Digestive System"). The multi-level system has four levels of groupings for diagnoses and we use the highest-order, most broad level to examine and assess disease groupings [18]. In our study, we used 281 mutually exclusive single-level, 18 level-one, and 89 level-two of multi-level categories (broader level) from CCS to map the disease categories based on their ICD-9-CM codes.

### Feature selection and statistical analysis

We used the Least Absolute Shrinkage and Selection Operator (LASSO) algorithm with Corrected Akaike Information Criterion (AICC) statistic (Eq 1)[19] for feature selection from all clinical variables. LASSO provides stability and robustness statistics that generate sparse models and thus can be used for L1 feature selection. LASSO seeks not only a well-fit model but also a “simple” one that will avoid the large variation that comes with estimating complex models [20]. We then used logistic regression for odds ratio estimates for satisfaction outcomes for each question. We used Euclidean distance matrix in pvclust R package[21] to calculate the similarity of the responses with 1,000 bootstrap replicates to adjust for false positives and false negatives. We used ANOVA and chi-square test to analyze continuous and categorical clinical variables, carried out using SAS 9.3.2 (SAS institute, Cary, NC) and R 2.15.1 [22]. Data was presented as mean ± standard deviation. *P*<0.05 was considered statistically significant.

### Network visualization

To evaluate the similarity of each question pairs (Q-Q) we computed a similarity matrix based on their significant shared clinical features. Similarity was estimated using the cosine distance for all combinations of question pairs. The q-value[23] to control for multiple-hypothesis testing and assess significance of similarity between each Q-Q. We randomly shuffled the clinical features across all the questions and re-computed the Q-Q distance. To reduce the false positive and false negatives, we repeated the randomization procedure 1,000 times to estimate the null distribution of the cosine distance for each Q-Q pair. The q-values were calculated as the ratio of the expected number of false positives over the total number of hypotheses tested [23]. A q-value ≤ 0.01 was chosen as a significant association threshold between Q-Q pairs. We used Cytoscape 3.2.0[24] to visualize the networks for the Q-Q pairs identified as significant and the risk factor-question significant associations.

$$AICC=1+\text{ln}\left(\frac{SSE}{n}\right)+\frac{2\left(k+1\right)}{n-k-2}$$ Eq 1

where *k* is the number of parameters in the model, and *n* is the sample size.

$$\cos ine-similarity(Q,Q)=\frac{Q•Q}{\left\|Q\right\|\left\|Q\right\|}=\frac{\sum_{i=1}^{n}Q_{i}\times Q_{i}}{\sqrt{\sum_{i=1}^{n}{\left(Q_{i}\right)}^{2}}\times \sqrt{\sum_{i=1}^{n}{\left(Q_{i}\right)}^{2}}}$$ Eq 2

Where *Q* is the question and *i* is the risk factor

## Results

### Patient characteristics

The survey data comprised 1,002 (57%) females and 769 (43%) males. The average participant age was 63 years for female, male, and the overall survey population. Survey responders were predominantly collected from twenty-one urban New York Counties with a self-reported ethnicity distribution of: 46.81% White, 24.22% African American, 2.6% Asian, 0.23% Native American/Eskimo, and 26.14% Other. Among those that self-reported as Other, 49.68% patients reported as Puerto Rican origin or descent. We collected 28 characteristics variables for responders including: demographics, admission/discharge information, primary diagnosis/procedure, secondary diagnosis/procedure, insurance plan, cost expense, and mortality risk, all of which are detailed in S3 Table. Age, gender, and race were matched for the surveyed patients from 2010 to 2012. Number of total diagnosis, procedures, and comorbidities were significantly lower in 2012 compared to earlier years. Admission source from emergency room (ER) was exclusively present only in 2010 as this resource was recorded prior to July 2010. The amount of individuals discharged to their home under the care of an organized home health service was significantly fewer in 2011 compared to other years. In 2012, more patients were on Medicare indemnity and Medicare insurance plans (S2 Table) than other plans.

### Concordance of the responses from same questionnaire category

**Fig 1:**
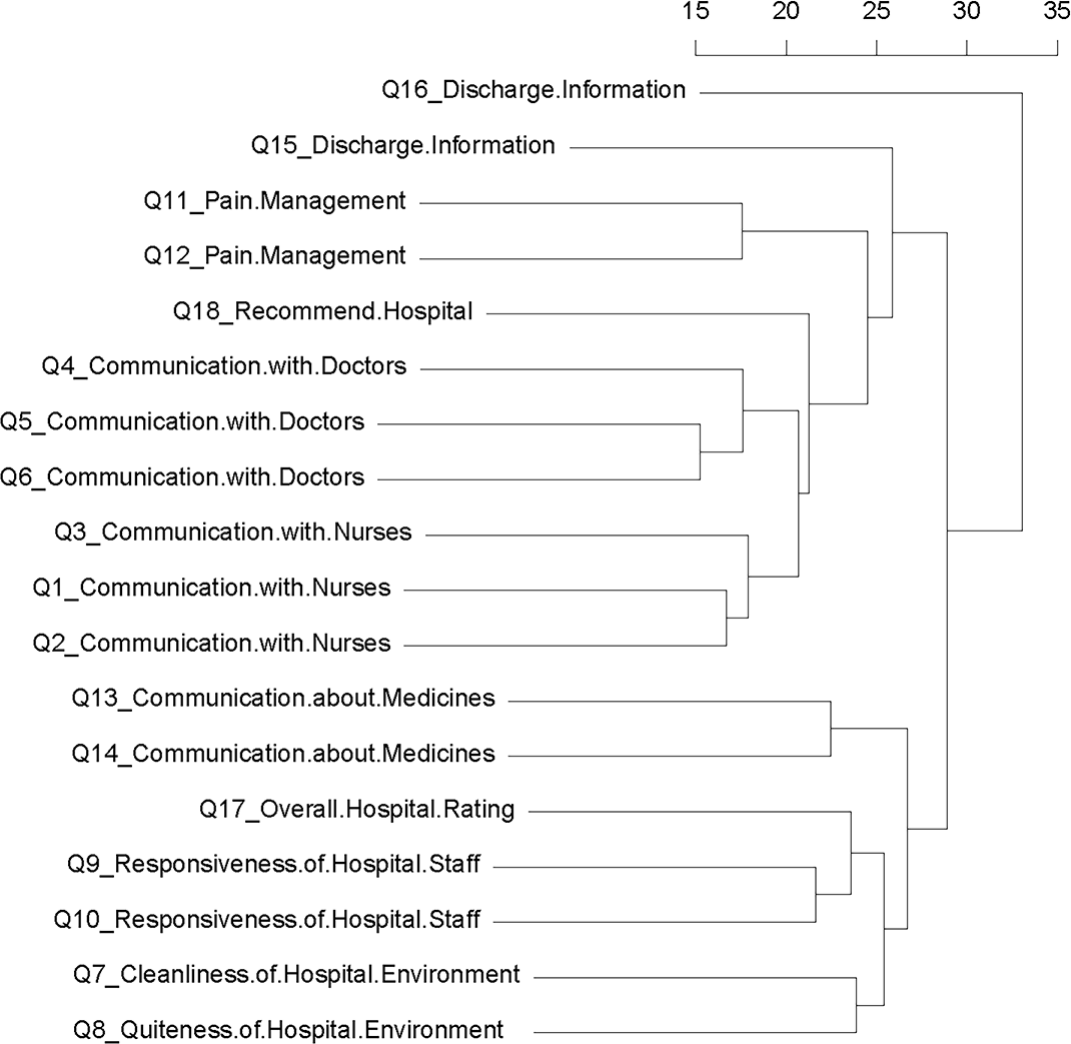
Hieratical clustering on binary responses for 18 questions. The cluster consists of similarity of 18 questions (see Methods).

We first evaluated the similarity of the binary responses to ensure the accurate collection procedures across all 18 questionnaires by hierarchical clustering. Among the 6 composite categories, all questions within the same categories (S1 Table) were clustered together, including ‘communication with nurses’ (Q1-3), ‘communication with doctors’ (Q4-6), ‘responsiveness of hospital staff’ (Q9-10), ‘pain management’ (Q11-12), ‘communication about medicines’ (Q13-14). ‘discharge information’ (Q15-16) clustered outside the main cluster (Fig 1). Two individual topics on ‘cleanliness of hospital environment’ (Q7) and ‘quietness of hospital environment’ (Q8) were clustered tightly together. The two global topics, ‘overall hospital rating’ (Q17) was co-clustered with ‘responsiveness of hospital staff’ and ‘hospital recommendation’ (Q18) was co-clustered with both ‘communication with doctors’ and ‘communication with nurses’. Nevertheless, response data clustering is robust except for question (Q16) on ‘discharge information’ (Fig 1).

### Risk factors associating with six composite topics

#### Communication with nurses

We identified 16, 13, and 14 risk factors that significantly associated with Q1, Q2, and Q3 (Table 1, S3 Table), respectively. Of the 43 total risk factors, 33 risk factors were unique, and 3 risk factors were shared among all three questions: self-evaluation of health condition, education level, and Asian. The better patients self-evaluated their health, the better communication they had with nurses (OR: 1.36 [1.23–1.51], 1.3 [1.19–1.42], and 1.29 [1.18–1.42] for Q1- 3 respectively). In contrast, patients with higher education levels (OR: 0.84 [0.78–0.91], 0.85 [0.79–0.91], 0.89 [0.83–0.95] for Q1-3 respectively), or patients who identified themselves as Asian were less likely to be satisfied with their communication with nurses (OR: 0.19 [0.1–0.37], 0.31 [0.16–0.59], 0.35 [0.19–0.67] for Q1-3 respectively). Additionally, four risk factors were significantly associated with at least 2 questions regarding communication with nurses: ‘*Other disorders of stomach and duodenum*’ as primary diagnosis, pregabalin medication order, treatment in bone marrow transplant (BMT) oncology division, and African American race. Patients were least likely to be satisfied if they had a primary diagnosis of ‘*Other disorders of stomach and duodenum’* (OR: 0.34 [0.13–0.91] for Q1, 0.24 [0.08–0.68] for Q2) or were prescribed pregabalin, a neuropathic pain relief medication (OR: 0.14 [0.04–0.51] for Q1 and 0.2 [0.05–0.81] for Q3). However, patients who were treated in the BMT oncology division (OR: 3.76 [1.79–7.89] for Q1 and 2.26 [1.25–4.06] for Q2) or were African American (OR: 1.68 [1.22–2.31] for Q1, and 1.78 [1.35–2.35] for Q3) were more likely to be satisfied with their communication with nurses.

**Table 1:** Top Ranked Risk Factors for Each Category and Directions.

Risk Factors% (Num)	CMC.w.Nurses (Q1-3)	CMC.w.Drs(Q4-6)	RS.of.Staff (Q9-10)	Pain.Mgmt (Q11-12)	CMC.Meds (Q13-14)	DC.Info. (Q15-16)	Hospital.Env. (Q7-8)	Overall.Rating (Q17)	Rec.Hospital (Q18)	Total	Pos. or Neg.
cms.self.e.health	100 (3)	100 (3)	100 (2)	100 (2)	100 (2)	-	100 (2)	100 (1)	100 (1)	89 (16)	+
cms.education	100 (3)	100 (3)	100 (2)	-	100 (2)	50 (1)	100 (2)	100 (1)	100 (1)	83 (15)	-
race.asian	100 (3)	67 (2)	50 (1)	100 (2)	50 (1)	-	100 (2)	-	100 (1)	67 (12)	-
race.white	33 (1)	-	-	100 (2)	50 (1)	-	100 (2)	100 (1)	100 (1)	44 (8)	-
div.bmt.oncology	67 (2)	-	50 (1)	-	50 (1)	-	100 (2)	-	100 (1)	39 (7)	+
cms.new.med	33 (1)	67 (2)	-	50 (1)	-	-	100 (2)	100 (1)	-	39 (7)	-
admis.status.er	33 (1)	100 (3)	-	-	50 (1)	50 (1)	-	100 (1)	-	39 (7)	-
dis.status.home	33 (1) +	33 (1) +	50 (1) +	-	50 (1) +	50 (1) -	-	-	100 (1) +	33 (6)	+/-
dx.other.dis.stomach	67 (2)	100 (3)	-	50 (1)	-	-	-	-	-	33 (6)	-
religion.catholic	-	67 (2)	-	50 (1)	-	-	50 (1)	-	100 (1)	28 (5)	+
race.aa	67 (2)	33 (1)	-	-	-	-	50 (1)	-	-	22 (4)	+
cms.home	-	67 (2)	-	50 (1)	-	-	-	-	100 (1)	22 (4)	+
rem.riskmodel	33 (1)	-	100 (2)	50 (1)	-	-	-	-	-	22 (4)	-
rx.pregabalin	67 (2)	-	-	100 (2)	-	-	-	-	-	22 (4)	-
cms.dis.facility	33 (1)	33 (1)	-	50 (1)	-	-	-	100 (1)	-	22 (4)	-
rx.pneumococcal.vac	33 (1)	100 (3)	-	-	-	-	-	-	-	22 (4)	-
dx.infec.dz.2nd	33 (1)	-	50 (1)	-	50 (1)	-	50 (1)	-	-	22 (4)	-
dx.ccs1.neoplasms	33 (1)	-	-	50 (1)	50 (1)	-	-	-	-	17 (3)	+
rx.oxycodone	33 (1)	-	-	50 (1)	-	-	-	-	100 (1)	17 (3)	-
admit.severity	33 (1) -	-	-	-	-	50 (1) +	-	-	-	11 (2)	+/-
lab.lymph.ahigh	-	100 (3)	-	-	-	-	-	-	-	17 (3)	-
los.obs	-	-	-	-	-	100 (2)	-	-	-	11 (2)	+
dx.ccs1.metabolic	-	67 (2)	-	-	-	-	-	-	-	11 (2)	-
cms.nonhispanic	-	-	-	50 (1)	50 (1)	-	-	-	-	11 (2)	-
uhc.2nd.commerial	-	33 (1)	-	-	50 (1)	-	-	-	-	11 (2)	-
dx.pneumonia	33 (1)	33 (1)	-	-	-	-	-	-	-	11 (2)	+
1proc.eye.or	33 (1)	33 (1)	-	-	-	-	-	-	-	11 (2)	-
num.cc	-	-	50 (1)	-	-	50 (1)	-	-	-	11 (2)	-
dx.diabetes	-	-	-	100 (2)	-	-	-	-	-	11 (2)	+
cms.puertorican	33 (1)	-	50 (1)	-	-	-	-	-	-	11 (2)	+
lab.sodium.alow	-	33 (1)	-	-	-	-	-	-	100 (1)	11 (2)	+
rx.loratadine	-	67 (2)	-	-	-	-	-	-	-	11 (2)	-
cms.help.bath.bed	-	-	50 (1)	-	-	50 (1)	-	-	-	11 (2)	+
lab.cr.ahigh	-	-	-	50 (1)	50 (1)	-	-	-	-	11 (2)	-
dx.viral.infection	33 (1)	-	-	-	50 (1)	-	-	-	-	11 (2)	-
religion.jewish	-	-	50 (1)	-	-	50 (1)	-	-	-	11 (2)	-
uhc.primary.hmo	-	33 (1)	-	-	-	-	-	100 (1)	-	11 (2)	-
1proc.cardio.or	-	-	-	-	-	-	50 (1) +	100 (1) -	-	11 (2)	+/-

#### Communication with doctors

We identified 14, 16, and 18 risk factors that significantly associated with Q4, Q5, and Q6 (Table 1, S3 Table), respectively. Forty risk factors were unique and six risk factors were consistently significant across all three questions: self-evaluation of health condition, education level, pneumococcal vaccine administration, admission through the emergency room (ER), primary diagnosis of ‘*other disorder of stomach and duodenum*’, and abnormally high lymphocyte counts. Similar to ‘communication with nurses’, the better patients self-evaluated their health, the better they perceived their communication with doctors (OR: 1.28 [1.15–1.43], 1.34 [1.22–1.48], and 1.34 [1.22–1.48] for Q4- 6 respectively). However, patients were again less likely to be satisfied if they reported a higher education level (OR:0.78 [0.72–0.85], 0.81 [0.75–0.87], 0.86 [0.81–0.92] for Q4-6 respectively), were administered the pneumococcal vaccine (OR: 0.4 [0.19–0.83], 0.39 [0.19–0.8], 0.43 [0.21–0.88] for Q4-6 respectively), had an abnormally high percentage of lymphocytes (OR:0.61 [0.41–0.92], 0.63 [0.43–0.91], 0.66 [0.45–0.95] for Q4-6 respectively), were admitted through the emergency room (ER) (OR: 0.46 [0.33–0.65], 0.61 [0.45–0.83], 0.69 [0.53–0.91] for Q4-6 respectively), or were primarily diagnosed with ‘*other disorder of stomach and duodenum*’ (OR: 0.26 [0.09–0.73], 0.22 [0.08–0.62], 0.33 [0.12–0.91] for Q4-6 respectively). Moreover, six additional risk factors were significantly associated with at least 2 questions related to physician communication: offered new medications, prescribed loratadine, Asian, diagnosed with “metabolic disease”, Catholic religion, and discharged to home. Patients were less satisfied if they were: offered new medications they had not previously taken (OR: 0.68 [0.53–0.86] for Q5, 0.69 [0.55–0.87] for Q6), taking loratadine OR: 0.33 [0.13–0.79] for Q5, 0.38 [0.16–0.88] for Q6), Asian (OR: 0.32 [0.17–0.62] for Q4, 0.4 [0.21–0.76] for Q6), or diagnosed with metabolic disease (OR: 0.56 [0.36–0.87] for Q5, 0.58 [0.38–0.9] for Q6). “Metabolic Disease” is a non-specific diagnosis defined by AHRQ that encompasses several disease states including: dehydration, diabetes with other specified manifestations, etc.[18] Alternatively, patients were more likely to be satisfied if they were of Catholic religion (OR: 1.36 [1.01–1.84] for Q4, 1.37 [1.06–1.78] for Q5) or were discharged directly to their home (OR: 1.53 [1.07–2.17] for Q4, 1.54 [1.13–2.1] for Q6) compared to those of other stated religions or those discharged to other destinations.

#### Responsiveness of hospital staff

We identified 10 and 12 risk factors significantly associated with Q9 and Q10, respectively (Table 1, S3 Table). Three of the twenty-two total risk factors were repeatedly significant for both questions: self-evaluation of health condition, education level, and relative expected mortality. Similar to what had been observed previously, the better patients self-evaluated their health, the more likely they were to be satisfied (OR: 1.44 [1.25–1.66], 1.26 [1.14–1.39] for Q9-10 respectively). Patients were less likely satisfied if they were in a higher education level bracket (OR: 0.86 [0.78–0.94], 0.82 [0.76–0.88] for Q9-10 respectively) or had a higher relative expected mortality (OR: 0.85 [0.73–0.99], 0.89 [0.79–0.99] for Q9-10 respectively).

#### Pain management

We identified 15 and 12 risk factors that significantly associated with Q11 and Q12, respectively. (Table 1, S3 Table). Five of the twenty-three unique risk factors were consistently significant for both questions: self-evaluation of health condition, Asian, White, pregabalin prescription, and ‘*diabetes with complications’* as primary diagnosis. Similarly, the better patients self-evaluated their health, the more likely they were satisfied with their pain management (OR: 1.19 [1.06–1.32], 1.19 [1.06–1.34] for Q11-12 respectively). Interestingly, patients diagnosed with “*diabetes with complications*” were also more satisfied with how their pain was managed (OR: 2.65 [1.03–6.82], 3.07 [1.06–8.9] for Q11-12 respectively). Patients were less likely to be satisfied if they were prescribed pregabalin (OR: 0.16 [0.03–0.79], 0.18 [0.05–0.68] for Q11-12 respectively), were Asian (OR: 0.14 [0.05–0.41], 0.26 [0.11–0.62] for Q11-12 respectively), or were White (OR: 0.62 [0.47–0.81], 0.57 [0.41–0.79]).

#### Communication about Medicines

We identified 14 and 7 risk factors that significantly associated with Q13 and Q14, respectively, 19 of which were unique (Table 1, S3 Table). Two of the nineteen unique risk factors were consistently significant for both questions: self-evaluation of health condition and education level. Similarly, the better patients self-evaluated their health, the more likely they were satisfied (OR: 1.23 [1.09–1.39], 1.22 [1.1–1.36] for Q13-14 respectively). The higher the patient’s education level, the less likely they were to be satisfied (OR: 0.86 [0.79–0.95], 0.85 [0.78–0.92] for Q13-14 respectively).

#### Discharge information

We identified 12 and 9 risk factors that significantly associated with Q15 and Q16, respectively, 20 of which were unique. As the responses for both questions were not clustered together, we were only able to identify one common risk factor: observed length of hospital stay (S1 Fig, Table 1, and S3 Table). Specifically, the longer the patients stayed in the hospital, the more satisfied they were with their discharge information (OR: 1.04 [1.01–1.07], 1.1 [1.04–1.16] for Q15-16 respectively). Moreover, patients diagnosed with “*intestinal obstruction without hernia*” were least satisfied with Q15 (OR: 0.23 [0.06–0.87]), and patients taking acetaminophen were least satisfied with Q16 (OR: 0.14 [0.04–0.49]).

A complete list for each category above is detailed in Table 1, S3 Table, S4 Table, and S1 Fig.

### Risk factors associating with hospital environment

#### Cleanliness and quietness of hospital environment

We identified 13 and 11 risk factors that significantly associated with Q7 and Q8, respectively, 19 of which were unique (Table 1, S3 Table). Six risk factors were consistently significant for both questions: self-evaluation of health condition, education level, Asian, White, treatment in BMT oncology division, and offered new medications. The better the patients evaluated their own overall health condition, the happier they were with hospital environment (OR: 1.24 [1.13–1.35], 1.2 [1.11–1.31] for Q7-8 respectively). Patients were more likely to be satisfied if they were treated in the BMT oncology division (OR: 1.85 [1.16, 2.94], 2.35 [1.49, 3.69] for Q7-8 respectively). In contrast, patients were less likely to be satisfied if they had a higher education level (OR: 0.83 [0.77–0.88], 0.84 [0.79–0.9]), White (OR: 0.77 [0.61–0.97], 0.6 [0.46–0.79]), Asian, (OR: 0.27 [0.14–0.51], 0.39 [0.19–0.8), or if they were offered new medication not previously taken (OR: 0.74 [0.6–0.91], 0.7 [0.57–0.86] for Q7-8 respectively). A complete list for each category above is shown in Table 1, S3 Table, and S1 Fig.

### Risk factors associating with global topics

#### Overall hospital rating

We identified 11 risk factors significantly associated with overall hospital rating, two of which were positively related and nine of which were negatively related. County of origin was a significant driver of overall hospital rating with patients from Kings County more likely to give a higher score as compared to those from other counties in New York or out of state (OR: 1.49 [1.03–2.12]). Patients who self-reported a better health condition also gave a higher overall hospital rating (OR: 1.36 [1.25–1.49]). Patients were less likely to give a higher rating if they: were diagnosed with urinary tract infections (OR: 0.19 [0.05–0.67], had abnormally low mean corpuscular hemoglobin levels (OR: 0.39 [0.17–0.93]), underwent cardiovascular system procedures (OR: 0.44 [0.25–0.77]), were discharged to a facility (OR:0.48 [0.3–0.78]), White (OR: 0.57 [0.42–0.72]), had an HMO as their primary insurance payer (OR: 0.58 [0.42–0.79]), were admitted through the ER (OR: 0.61 [0.47–0.79]), were offered a new medication not previously taken prior to hospitalization (OR: 0.71 [0.59–0.9]), or had a higher level of education (OR: 0.77 [0.72–0.82]) (Table 1, S3 Table, S1 Fig).

#### Hospital recommendation

Similar to what was observed with overall hospital rating, we identified 11 risk factors significantly associated with willingness to recommend, of which only two risk factors (self-reported health and White race) were shared. Patients were more likely to recommended the hospital if they were treated in the BMT oncology division (OR: 2.48 [1.38–4.44]), had abnormally low sodium levels (OR: 1.56 [1.23–1.97]), were discharged to home as per CMS broad measure (OR:1.52 [1.11–2.07]) or hospital-wide detailed measure (OR:1.41 [1.11–1.81]), were of Catholic religion (OR: 1.3 [1.02–1.67]), or self-reported a better health condition (OR: 1.22 [1.11–1.34]). Moreover, patients were less likely to recommended the hospital if they were prescribed emtricitabine/tenofovir, a fixed-dose combination medication of two antiretroviral drugs used to treat HIV (OR: 0.19 [0.05–0.83]), Asian (OR: 0.28 [0.15–0.54]), prescribed oxycodone/acetaminophen to treat moderate to moderately severe pain (OR: 0.29 [0.16–0.54]), White (OR: 0.62 [0.48–0.79]), or if they had a higher level of education (OR: 0.89 [0.83–0.96]) (Table 1, S3 Table, S1 Fig).

### Top ranked risk factors across all topics

**Fig 2:**
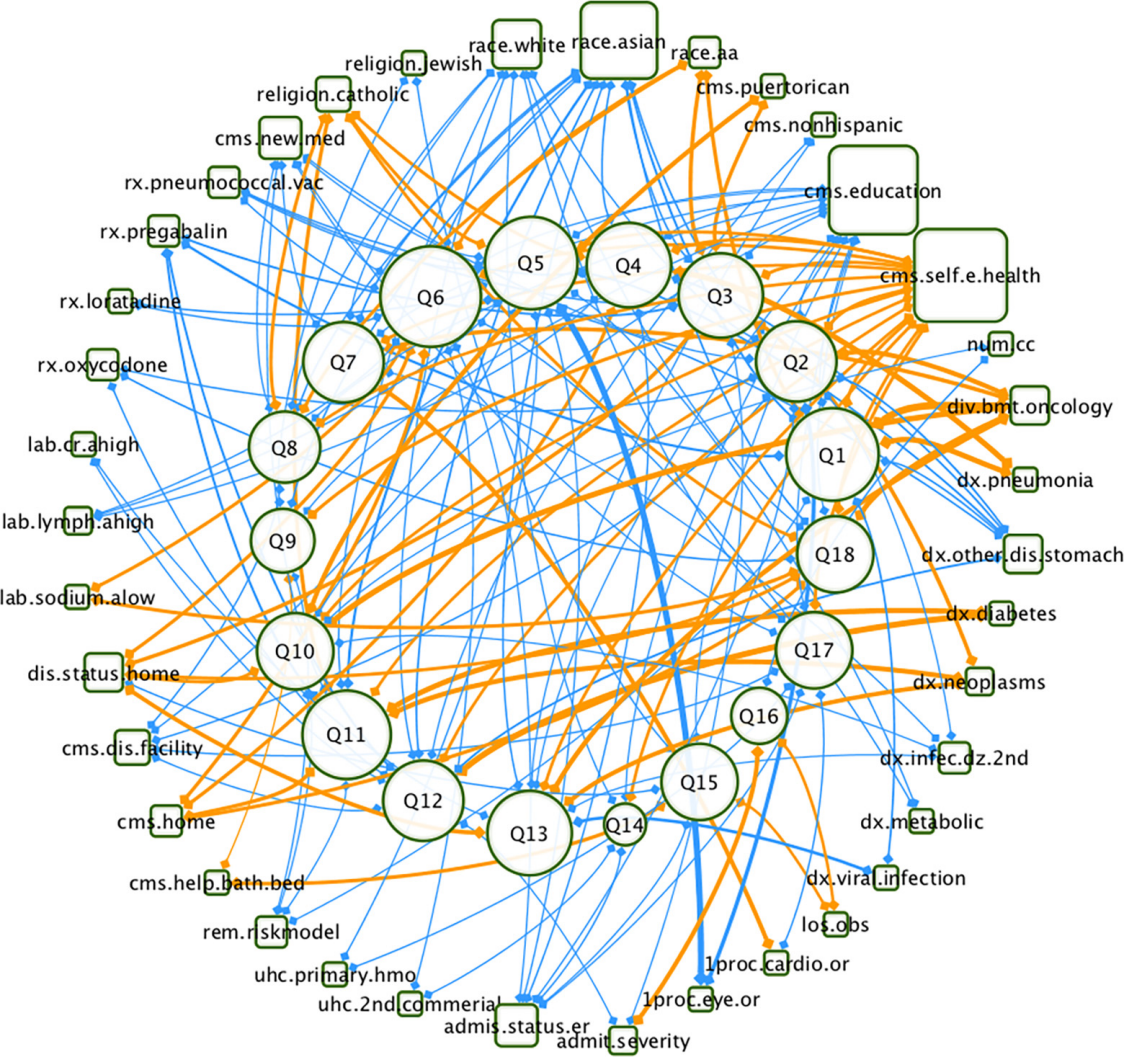
Network of risk factors (selected more than once in risk model profile) to each question. The network consists of the 38 risk factors, which have been selected more than once in risk model profile (Table 1, S3 Table) and 18 questions (S1 Table) with the LASSO algorithm. Risk factors (blue rectangle) and questions (orange circles) are connected by green lines (negative association) or red lines (positive association). The node size reflects the amount of associated risk factors. The edge width and the color grade reflect strength of the odds ratio from the risk model. This network was visualized using Cytoscape 3.2.0.

Across the 102 unique significant risk factors to all 18 questions, 38 risk factors were consistently identified for more than one question. Except for 3 risk factors that had mixed association directionality, of the remaining 35 risk factors, 12 risk factors were always positively associated with patient satisfaction while 23 risk factors were always negatively associated with patient satisfaction (Table 1, S3 Table, and Fig 2). The most frequently identified negative risk factors, associated with more than half of all 18 questions, were as follows: poor self-evaluation of health (16/18, 89%), higher education level (15/18, 83%), and Asian ethnicity (12/18, 67%), which are all consistent with what has been reported in previous studies [25–27]. White individuals were overall less satisfied (8/18, 44%), and African American individuals were generally more satisfied (4/18, 22%) particularly in the questions related to *communication with nurses* (2/3, 67%).

Patients diagnosed with ‘*Other disorders of stomach and duodenum’* were least satisfied amongst all diagnoses (6/18, 33%) especially in regards to *communication with doctors* (3/3, 100%), *communication with nurses* (2/3, 67%), and *pain management* (1/2, 50%). However, patients diagnosed with any type of neoplasm were, on average, the more satisfied (4/18, 22%), primarily in regards to issues surrounding *pain management* (1/2, 50%) and *communication about medicines* (1/2, 50%). Patients diagnosed with diabetes were more satisfied with *pain management* (2/2, 100%); however, patients prescribed pregabalin were less satisfied compared to patients prescribed other medications (4/18, 22%) particularly in relation to *communication with nurses* (2/3, 67%) and *pain management* (2/3, 100%). Interestingly, patients who were administered the pneumococcal vaccine and patients with abnormally high lymphocyte levels were less satisfied, predominantly with *communication with doctors* (3/3, 100%). More details are shown in Table 1 and network Fig 2.

### Topic similarities based on their common risk factors

**Fig 3:**
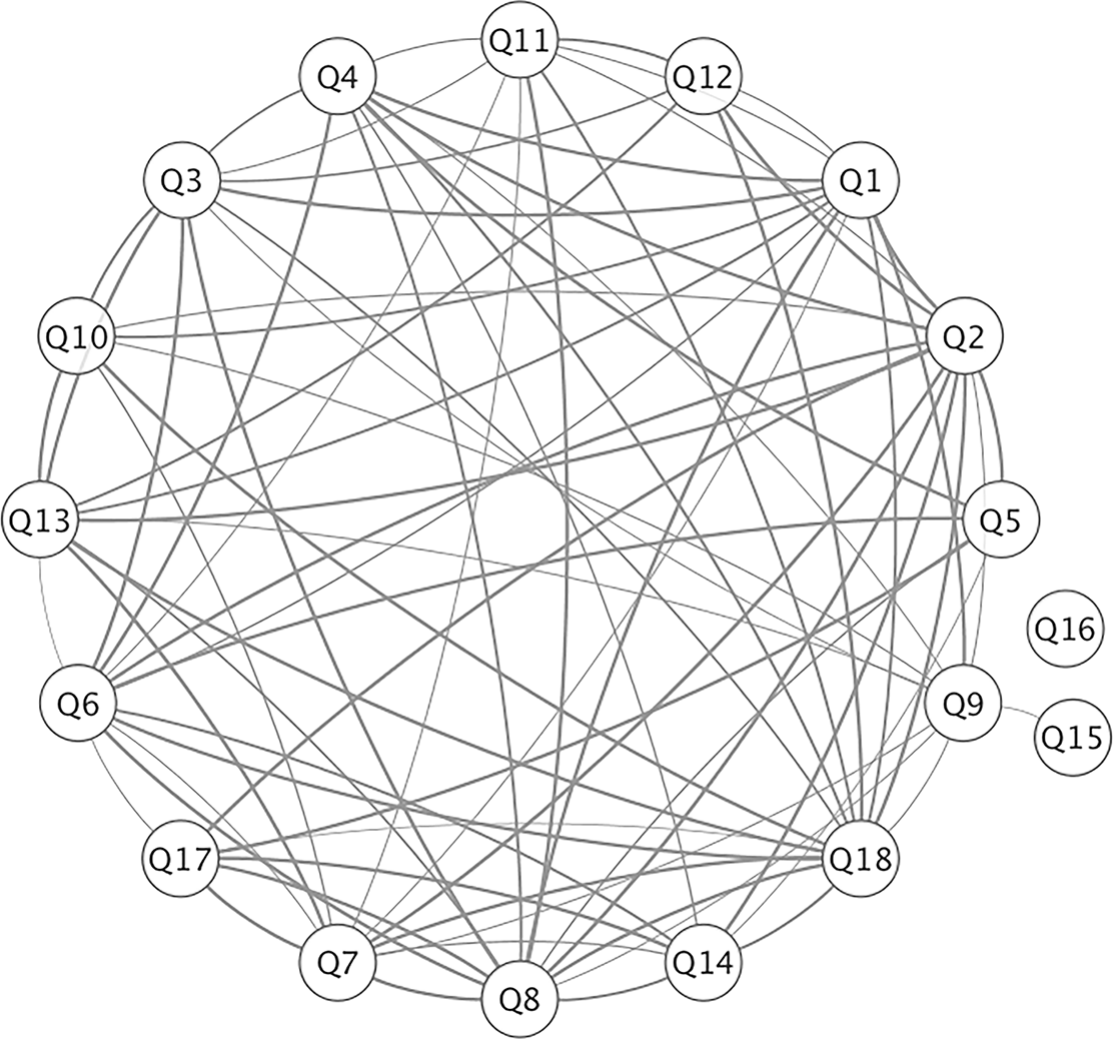
Q-Q similarity network based on common risk factors. The network consists of the 18 questions connected to each other with a criterion of q ≤ 0.01. Questions (circles) are connected by gray lines. The width of the edge reflects the significance of the q value. This network was visualized by Cytoscape 3.2.0.

We created a network visualization to evaluate the similarity of question-question (Q-Q) pairs where questions (nodes) are connected by an edge based on their shared risk factors. We identified 80 Q-Q pairs with q-value ≤ 0.01 and incorporated them into a network Fig 3. Except for the *communication about medicines* (Q13-14) and *discharge information* (Q15-16) topics, all questions in the same topics were associated, indicating they shared significant common risk factors. Interestingly, Q13, a question involving how well medication use was explained, was associated with all questions related to *communication with nurses* (q = 0.001, Q1-3), but Q14, a question involving how well the side effects of a medication were explained, was associated with all questions related to *communication with doctors* (q = 0.003, Q4-6). This suggests that specific components related to communication about medicines influence a patient’s perception of how well they communicate with specific providers (i.e., nurse versus doctor). *Overall hospital rating* (Q17) was significantly impacted by *hospital environment* (Q7-8, 100%), *communication with doctors* (Q5-6, 67%), and *communication about medicines* (Q14, 50%). Moreover, *hospital recommendation* (Q18) was related to all of the HCAHPS questions specifically on *hospital environment* (Q7-8, 100%), *communication about medicines* (Q13-14, 100%), *pain management* (Q11-12, 100%), and *communication with nurses* (Q1-3, 100%) (Fig 3). In contrast, Q16, a question regarding whether or not the patient received information in writing regarding potentially worrisome symptoms or health problems to look out for after discharge, was not significantly connected to any of the other HCAHPS questions and as such is likely a less desirable area for improvement efforts (Fig 1).

## Discussion

We utilized a data-driven, machine learning approach for identifying factors associated with patient satisfaction during an inpatient hospital stay. The goal was to identify specific drivers of patient experience as measured by the HCAHPS survey questions so as to discovery novel, targeted corrective actions to better improve patients’ experience of care. We aspire to enact more precise, predictive, and personalized aspects of care by identifying the factors that are associated with low patient satisfaction in a specific hospital environment.

We identified 102 unique significant risk factors to all 18 questions in the HCAHPS survey including 38 risk factors consistently presented in more than one question. The most recurrent clinical features associated in a consistent direction (positive or negative) with patient satisfactions include: self-evaluation of health, education level, Asian, White, treatment in BMT oncology division, being prescribed a new medication, ER as admission status, diagnosis of “other disorders of stomach and duodenum”, and catholic as self-reported religion status. An immediate goal of the current study is to demonstrate how hospitals may create a better experience of care for their patients by identifying these factors within their own population of patients. Using this approach, hospitals can develop a system to proactively identify specific patients in specific clinical situations who are at increased risk for a negative patient experience so that they may better address these patients’ needs and improve their experience of care. While this may include an increased emphasis on communication on the part of the patient’s nurses and physicians during the inpatient stay, interventions may also include enhanced medication reconciliation by a clinical pharmacist or more active referral to a patient representative or clergy, depending on the need. Such proposed interventions may require increased hospital resources but will more likely require better utilization of existing ones. Four prescribed medications were all negatively associated with satisfaction (S3 Table), including pregabalin, which was inversely associated with pain management and communication with nurses. While it is important to consider alternative medications to opioids for the management of pain, based on our results, it is possible that pregabalin did not effectively control pain in the inpatient setting. This may be the result of providers attempting to curb the use of opioids in patients who consider opioids the standard for pain control and therefore expect and demand such therapy. It may also be related to the relative new use of pregabalin to treat acute pain, particularly post-operatively. Given pregabalin is often used to treat patients with neuropathies that are frequently difficult to manage clinically, the negative association between pregabalin and pain management and nursing communication could simply represent a more challenging patient population that requires increased attention to symptom management.

In order to understand the architecture of shared risk factors among questions, we constructed a Q-Q network and assessed the question similarities based on the significantly shared clinical features. In this way we can better understand the complex relationships between risk factors and dimensions of the survey questions in a data-driven manner. We discovered that explaining drug usage to patients was associated with *communication with nurses*; however, explaining drug side effect was primarily associated with *communication with doctors*. This suggests that patients prefer to receive information about their care from different providers. In addition, we identified that *o*v*erall hospital rating* was predominantly associated with *hospital environment*, *communication with doctors*, and *communication about medicines*. This suggests that in order to improve overall hospital rating, hospital leaders need to focus on these three composite questions.

Our analysis identified several risk factors previously associated with patient satisfaction. For instance, patients who poorly evaluated their health, had a higher education level, or were Asian were more likely to be dissatisfied with their hospital experience [25–27]. Previous studies have demonstrated lower patient satisfaction scores in Asians without clearly identifying what underlying factors drive their tendency to respond less favorably as compared to patients of other races and ethnicities.[26] However, in our study, we find that despite similar in levels of education (23% with 4 years college, 37% with graduate school), Asian patients were largely less satisfied with both *communication with nurses*, *communication with doctors*, and *responsiveness of staff* as compared to White patients. This could reflect cultural differences or suboptimal communication between patients and providers related to limited English proficiency. Given that the current hospital workflow relies on nurses to communicate most frequently with patients regarding their medications and other aspects of their care, it is important that translation services be readily accessible to allow optimal communication.

Our analysis also identified geographic risk factors in patient satisfaction that may underlie socioeconomic factors. We find that patients from Kings County were more likely to give a higher hospital rating. Data from the United States Census Bureau data demonstrates that, on average, residents in Kings County represent the lowest socioeconomic class in New York state with a median household income of $46,085 (versus a median household income of $58,003 for the state of New York). In addition, the high school graduation rate is lower in Kings County as compared to other counties in New York State (78.5% vs. 85.2%, respectively) (http://quickfacts.census.gov). This may suggest differences regarding expectations in patients of lower socioeconomic class and lower levels of education result in different perceptions of what comprises a positive patient experience, or may represent differences in care coordination for hospitalized patients at increased need.

Our findings reaffirm the importance of the hospital environment in patient satisfaction. For example, if the environment is perceived as unclean or unkempt, hospitals may consider reallocating environmental services; however, if the environment is perceived negatively because of the excessive noise, increased attention to “quiet time” and better level-setting regarding patient expectations on admission may be considered. Lastly, patients who would highly recommend the hospital to others were most likely to consider *hospital environment*, *communication about medicines*, *pain management*, and *communication with nurses*. An increased focus on these factors may better allow hospitals to specifically increase scores related to likelihood to recommend. This is an important dimension bearing on the overall perception of the hospital and its ability to maintain and grow its patient population.

Integration of 266 clinical features from high-dimensional EMR data is a unique aspect of our approach that identified new opportunities for understanding patient satisfaction. To minimize the false positives and false negatives, we used bootstrapping procedure with a stringent q-value to build the topic network and mainly focus on the risk factors that were associated at least with two topics. Additional clinical variables may help to better define or pinpoint patients or clinical contexts for which more precise efforts towards improving patient satisfaction can be directed. This provides a tractable framework that enables initial steps towards improving quality of patient care through clinical risk management. Our analysis identified a number of putatively novel clinical risk factors for patient satisfaction, which suggest new opportunities to better understand and manage patient satisfaction. This approach of leveraging the high dimensional EMR demonstrates a potential use of this methodology and proposes the utility for other hospitals striving to identify the underlying clinical risk factors for an effective inpatient care.

We identified several novel clinical risk factors pertaining to communication with healthcare practitioners. While the survey topics are similar for nurses (Q1-3) and doctors (Q4-6), we identified risk factors that were specific between the two, potentially highlighting unique opportunities in redesigning how providers communicate with certain patients within the hospital setting. We identified risk factors that associated specifically to communication with doctors, such as being offered a new medication, being prescribed loratadine, being given a pneumococcal vaccine, being admitted through the ER, self-reporting a religion other than Catholicism, being discharged to a destination other than home, having a diagnosis of “metabolic disease”, or having abnormal high lymphocyte lab measures. In contrast, we identified that being prescribed pregabalin was a negative risk factors specifically associated with communication with nurses. Understanding how and why these risk factors influence or correlate with an individual patient’s perception of how well they communicate with their physician or nurse would enact measures to ensure good communication prior to discharge. Lastly, being admitted through the ER was a shared risk factor between *communication about drug side effects* (Q14) and *communication with doctors* (Q4-6) (Fig 3). Perhaps the fast-paced environment of the ER leads to breakdowns in good communication between providers and patients.

We identified three clinical features exhibiting bi-directionality in predicting patient satisfaction: discharge to home, admission severity, and cardiovascular operation as first procedure (Table 1). For example, an outcome of ‘discharge to home’ was negatively associated with the survey question regarding discharge information (Q15: “Did hospital staff talk with you about whether you would have the help you needed when you left the hospital?”). However, ‘discharge to home’ was positively associated with other survey questions, including: *communication with nurses*, *communication with doctors*, *responsiveness of hospital staff*, *communication about medicines*, and *willingness to recommend*. This may suggest that although patients discharged to home are generally satisfied with the level of communication during their inpatient stay, providers may not be adequately addressing post-acute care needs. In addition to its impact on patient experience, failure to properly coordinate care in a subset of patient after discharge is significant because it can result in a suboptimal transition of care that could lead to avoidable morbidity and unnecessary readmission.

In this study, we used disease classification categories for presenting data at a descriptive statistic categorical level rather than using individual ICD-9-CM codes to associate disease status with survey responses. When stratified by higher-level disease categories, diagnoses of “neoplasms”, *“*pneumonia”, and “diabetes” were positively associated with patient satisfaction; conversely, “other disorders of stomach and duodenum”, “secondary infectious diseases”, “metabolic diseases”, and “viral infection” were negatively associated with patient satisfaction. Similar to what is currently being done to improve hospital throughput and reduce avoidable readmissions, developing disease-specific clinical pathways that focus on patient satisfaction or incorporating such measures into existing clinical pathways may be ideal strategies to address predicted low patient satisfaction in patients with diagnoses negatively associated with the experience of care.

Sample size is a potential limitation of this study. Due to the relatively low average response rate across 2010–2012 (33%) we were unable to recruit as many patients as would be ideal from the entire in-hospital population. Furthermore, patient demographics were not fully matched across the three years specifically in number of total diagnosis, procedures, and complications (p<0.0001, S2 Table) which were significantly fewer in recent years. Our statistical approach adjusted for these disparities in clinical features and none were significantly associated with satisfaction in healthcare.

Although concerns have been raised regarding the current emphasis on measurement and management of patient satisfaction, reported associations between greater patient satisfaction and increased mortality and healthcare cost are largely related to studies of the ambulatory patient care experience and measures of outpatient physician communication [28]. The risk of increased cost and iatrogenic morbidity and mortality that results from the improper prescription of discretionary tests and treatment is likely greater in the outpatient setting where primary care physicians have limited time to discuss and challenge patient expectations. Also, primary care physicians may feel pressured to comply with patient requests in order not to lose them to a more accommodating provider. While such dynamics certainly exist to a degree in the inpatient setting, the acute care delivered during a hospitalization is increasingly directed by hospitalists who can reduce utilization and prevent unnecessary costs and avoidable harm by effectively communicating the plan of care and thereby transitioning patients and families to a safer, less costly care environment.

## Conclusions

We developed and applied an integrative, data-driven approach to better understand the complexity of patient satisfaction survey outcomes in a large academic medical center. We leveraged high-dimensional EMR data integrated with patient satisfaction survey outcomes to identify and investigate clinical features associating with patient satisfaction. We were successfully able to identify common and individualized risk factors to particular aspects of patient experience. We developed a question similarity (Q-Q) network that visualized and enabled interrogation of higher-dimensional similarity patterns among satisfaction survey questions based on broader consideration of clinical risk factors associating with each question. These findings can inform the process of achieving hospital quality improvement and overall patient care. We suggest that our results illustrate the utility and promise of data-driven approaches in identifying potential risk factors affecting patient satisfaction. We hope other hospital healthcare systems will adopt and improve upon this methodology to identify aspects contributing to lower patient satisfaction and adapt to a more personalized level of care.

## Supporting Information

S1 Fig Complete network of all 102 risk factors to 18 questions. The network consists of the 102 unique significant risk factors to all 18 questions (S1 Table) with LASSO algorithm. Risk factors (rectangle) and questions (circles) are connected by blue lines (negative association) or orange lines (positive association). The node size reflects the amount of associated risk factors. The edge width reflects the strength of the odds ratio from the risk model. This network was visualized using Cytoscape 3.2.0.
(PDF)

S1 Table All 18 Surveyed Questions in 10 Categories. (DOCX)

S2 Table Overall Patient Characteristics and by Year. (DOCX)

S3 Table Risk factors associated with each questions. (PDF)

S4 Table Data Dictionary used in Network and Tables. (DOCX)
