# Supplementary material for: Data-Driven Identification of Risk Factors of Patient Satisfaction at a Large Urban Academic Medical Center
Source: PLoS One. 2016 May 26;11(5):e0156076. doi: 10.1371/journal.pone.0156076 (PMC4881910; doi:10.1371/journal.pone.0156076)
Supplement: S1 Fig — The network consists of the 102 unique significant risk factors to all 18 questions (S1 Table) with LASSO algorithm. Risk factors (rectangle) and questions (circles) are connected by blue lines (negative association) or orange lines (positive association). The node size reflects the amount of associated risk factors. The edge width reflects the strength of the odds ratio from the risk model. This network was visualized using Cytoscape 3.2.0. (PDF) [file pone.0156076.s001.pdf]

S1 Fig: Complete network of all 102 risk factors to 18 questions

The network consists of the 102 unique significant risk factors to all 18 questions (S1 Table) with LASSO algorithm. Risk factors (rectangle) and questions (circles) are connected by blue lines (negative association) or orange lines (positive association). The node size reflects the amount of associated risk factors. The edge width reflects the strength of the odds ratio from the risk model. This network was visualized using Cytoscape 3.2.0.

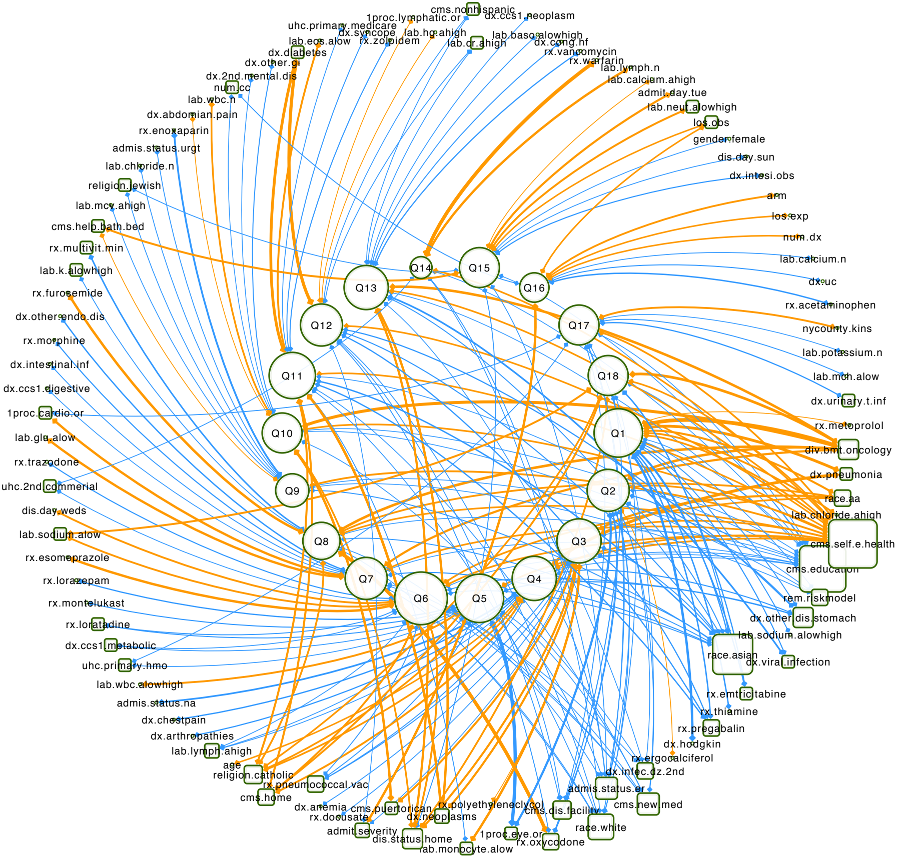
Q1
Q2
Q3
Q4
Q5
Q6
Q7
Q8
Q9
Q10
Q11
Q12
Q13
Q14
Q15
Q16
Q17
Q18
cms.nonhispanic
dx.ccs1.neoplasm
1proc.lymphatic.or
uhc.primary.medicare
dx.syncope
lab.hg.ahigh
lab.baso.alowhigh
rx.zolpidem
lab.eos.alow
lab.cr.ahigh
dx.cong.hf
dx.diabetes
rx.vancomycin
dx.other.gi
rx.warfarin
lab.lymph.n
dx.2nd.mental.dis
lab.calcium.ahigh
num.cc
admit.day.tue
lab.wbc.n
lab.neut.alowhigh
dx.abdomian.pain
los.obs
rx.enoxaparin
gender.female
admis.status.urgt
dis.day.sun
lab.chloride.n
dx.intesi.obs
religion.jewish
arm
lab.mcv.ahigh
los.exp
cms.help.bath.bed
num.dx
rx.multivit.min
lab.calcium.n
lab.k.alowhigh
dx.uc
rx.furosemide
rx.acetaminophen
dx.other.endo.dis
nycounty.kins
rx.morphine
lab.potassium.n
dx.intestinal.inf
lab.mch.alow
dx.ccs1.digestive
dx.urinary.t.inf
1proc.cardio.or
rx.metoprolol
lab.glu.alow
div.bmt.oncology
rx.trazodone
dx.pneumonia
uhc.2nd.commerial
race.aa
dis.day.weds
lab.chloride.ahigh
lab.sodium.alow
cms.self.e.health
rx.esomeprazole
cms.education
rx.lorazepam
rem.riskmodel
rx.montelukast
dx.other.dis.stomach
rx.loratadine
lab.sodium.alowhigh
dx.ccs1.metabolic
race.asian
uhc.primary.hmo
dx.viral.infection
lab.wbc.alowhigh
rx.emtricitabine
admis.status.na
rx.thiamine
dx.chestpain
rx.pregabalin
dx.arthropathies
dx.hodgkin
lab.lymph.ahigh
rx.ergocalciferol
age
dx.infec.dz.2nd
religion.catholic
admis.status.er
rx.pneumococcal.vac
cms.new.med
cms.home
rx.polyethyleneclycol
dx.anemia
cms.puertorican
cms.dis.facility
rx.docusate
dx.neoplasms
race.white
admit.severity
1proc.eye.or
dis.status.home
rx.oxycodone
lab.monocyte.alow
