# Supplementary material for: Data-Driven Identification of Risk Factors of Patient Satisfaction at a Large Urban Academic Medical Center
Source: PLoS One. 2016 May 26;11(5):e0156076. doi: 10.1371/journal.pone.0156076 (PMC4881910; doi:10.1371/journal.pone.0156076)
Supplement: S1 Table — (DOCX) [file pone.0156076.s002.docx]

**S1 Table: All 18 Surveyed Questions in 10 Categories**

| **Measures** | **Categories** | **Questions During Hospital Stay** |
| --- | --- | --- |
| Composite | Communication with Nurses | Q1. How often did nurses treat you with courtesy and respect? |
|  |  | Q2. How often did nurses listen to you carefully? |
|  |  | Q3. How often did nurses explain things in a way you could understand? |
|  | Communication with Doctors | Q4. How often did doctors treat you with courtesy and respect? |
|  |  | Q5. How often did doctors listen carefully to you? |
|  |  | Q6. How often did doctors explain things in a way you could understand? |
|  | Responsiveness of Hospital Staff | Q9. How often did you get help in getting to the bathroom or in using a bedpan as soon as you wanted? |
|  |  | Q10. After you pressed the call bell, how often did you get help as soon as you wanted? |
|  | Pain Management | Q11. How often was your pain well controlled? |
|  |  | Q12. How often did the hospital staff do everything they could to help you with your pain? |
|  | Communication about Medicines | Q13. Before giving you the meds, how often did hospital staff tell you what the medicine was for? |
|  |  | Q14. Before giving you the new meds, how often did hospital staff describe side effects in a way you could understand? |
|  | Discharge Information | Q15. Did hospital staff talk with you about whether you would have the help you needed when you left the hospital? |
|  |  | Q16. Did you get information in writing about what symptoms/health problems to look out for after you left the hospital? |
| Individual | Cleanliness of Hospital Environment | Q7. How often were your room and bathroom kept clean? |
|  | Quietness of Hospital Environment | Q8. How often was the area around your room quiet at night? |
| Global | Overall Hospital Rating | Q17. What number would you use to rate this hospital? (0: worst;10: best) |
|  | Recommend Hospital | Q18. Would you recommend this hospital to your friends and family? |
