# Supplementary material for: Data-Driven Identification of Risk Factors of Patient Satisfaction at a Large Urban Academic Medical Center
Source: PLoS One. 2016 May 26;11(5):e0156076. doi: 10.1371/journal.pone.0156076 (PMC4881910; doi:10.1371/journal.pone.0156076)
Supplement: S2 Table — (DOCX) [file pone.0156076.s003.docx]

**S2 Table: Overall Patient Characteristics and by Year**

|  |  |  |  |  |  |
| --- | --- | --- | --- | --- | --- |
| **Characteristics** | **Overall (n=1771)** | **2010 (n=360)** | **2011 (n=772)** | **2012 (n=639)** | ***P*** |
| Age | 63.16 ± 17.21 | 61.78 ± 17.74 | 63.4 ± 16.69 | 63.65 ± 17.5 | 0.23 |
| Gender (Female) | 57% | 57% | 57% | 56% | 0.81 |
| Race (White, Others, African American) | 47%, 26%, 24% | 46%, 26%, 26% | 49%, 24%, 23% | 44%, 29%, 24% | 0.39 |
| Ethnicity#* (Puerto Rican, Other, Non-His) | 50%, 24%, 15% | 55%, 26%, 9% | 55%, 24%, 13% | 41%, 23%, 18% | 0.03 |
| Religion* (Catholic, Unknown, Jewish) | 32%, 30%, 16% | 36%, 27%, 14% | 32%, 30%, 18% | 29%, 32%, 15% | 0.12 |
| NY County* (New York, Out of State, Bronx) | 51%, 10%, 9% | 54%, 8%, 10% | 50%, 12%, 9% | 50%, 8%, 9% | 0.45 |
| Number of Total Diagnosis | 10.28 ± 5.99 | 10.48 ± 5.61 | 10.86 ± 6.01 | 9.46 ± 6.09 | <.0001 |
| Number of Procedures | 0.78 ± 1.12 | 1.6 ± 2.09 | 0.58 ± 0.49 | 0.56 ± 0.5 | <.0001 |
| Number of Comorbidities and Complications | 7.46 ± 6.52 | 10.48 ± 5.61 | 10.86 ± 6 | 1.66 ± 1.98 | <.0001 |
| First Procecure Category* (Misc., Digestive Sys., Hemic/Lymphatic) | 25%, 13%, 6% | 31%, 11%, 45 | 25%, 14%, 6^ | 22%, 13%, 6% | 0.25 |
| Number of Secondary Diagnosis* |  |  |  |  |  |
| Endocrine, Nutritional & Metabolic Dz; Immunity Disorders | 1.31 ± 1.34 | 1.28 ± 1.3 | 1.35 ± 1.34 | 1.26 ± 1.37 | 0.43 |
| Diseases of the Circulatory System | 1.48 ± 1.65 | 1.61 ± 1.79 | 1.58 ± 1.7 | 1.29 ± 1.48 | 0.001 |
| Residual codes; Unclassified; all E codes | 1.46 ± 1.64 | 1.48 ± 1.52 | 1.54 ± 1.67 | 1.35 ± 1.65 | 0.1 |
| Secondary Procedures Category |  |  |  |  |  |
| External Injury | 0.34 ± 0.69 | 0.31 ± 0.7 | 0.4 ± 0.74 | 0.28 ± 0.63 | 0.01 |
| Division* (Hospitalist, Gastroenterology, Medical Oncology) | 51%, 15%, 13% | 44%, 18%, 14% | 49%, 16%, 12% | 56%, 12%, 13% | 0.07 |
| Admit Day* (Mon, Tues, Thur) | 18%, 17%, 16% | 19%, 17%, 13% | 18%, 17%, 19% | 13%, 19%, 14% | 0.02 |
| Admission Source* (NFPO^, ER, Hospital Transfer) | 84%, 8%, 4% | 55%, 38%, 3% | 90%, 0%, 4% | 94%, 0%, 4% | <.0001 |
| Admission Status* (Emergency, Urgent, Elective) | 76%, 14%, 9% | 76%, 13%, 11% | 73%, 15%, 11% | 80%, 13%, 6% | 0.01 |
| Admit Severity of Illness* (Moderate, Major, Minor) | 40%, 32%, 22% | 36%, 35%, 23% | 39%, 32%, 23% | 43%, 32%, 20% | 0.48 |
| Admit Risk of Mortality* (Minor, Moderate, Major) | 42%, 39%, 17% | 41%, 43%, 41% | 39%, 39%, 37% | 15%, 16%, 18% | 0.33 |
| Relative Expected Mortality* (2012 Risk Model; Below, Well Below, Above) | 54%, 32%, 11% | 54%, 32%, 10% | 54%, 31%, 12% | 54%, 32%, 11% | 0.95 |
| Discharge Day* (Fri, Weds, Thur) | 21%, 18%, 17% | 21%, 18%, 16% | 20%, 19%, 17% | 21%, 17%, 17% | 0.002 |
| Discharge Status* (Home, Home Under Care, Skilled Nursing Facility) | 71%, 21%, 5% | 68%, 26%, 4% | 75%, 17%, 5% | 68%, 24%, 5% | 0.02 |
| UHC Primary Payer* (Medicare Indemnity, Medicare, HMO) | 40%, 14%, 14% | 38%, 14%, 16% | 41%, 12%, 16% | 40%, 16%, 10% | <.0001 |
| UHC Secondary Payer* (Self-Pay, Medicaid Indemnity, Private Indemnity) | 32%, 24%, 18% | 36%, 24%, 18% | 33%, 24%, 18% | 30%, 24%, 18% | 0.05 |
| Length of Stay Expected (2012 Risk Model) | 5.46 ± 4.84 | 5.26 ± 4.64 | 5.46 ± 4.79 | 5.57 ± 5 | 0.63 |
| Length of Stay Observed | 6.14 ± 7.74 | 6.07 ± 7.19 | 6.04 ± 6.91 | 6.3 ± 8.9 | 0.81 |
| ICU Days Observed | 0.2 ± 1.83 | 0.33 ± 2.88 | 0.13 ± 0.98 | 0.21 ± 1.86 | 0.24 |
| Direct Cost Observed | 8474.74 ± 17068.76 | 7405.26 ± 12765.94 | 8993.79 ± 17822.03 | 8450.18 ± 18214.21 | 0.35 |
| Total Cost Observed | 13230.48 ± 22939.11 | 11813.43 ± 18953.09 | 13562.77 ± 22392.27 | 13627.36 ± 25501.88 | 0.42 |
| *: Ranked as top 3 incidences |  |  |  |  |  |
| ^: Non-Facility Point of Origin |  |  |  |  |  |
| #: self-reported race as others |  |  |  |  |  |
