# Supplementary material for: Data-Driven Identification of Risk Factors of Patient Satisfaction at a Large Urban Academic Medical Center
Source: PLoS One. 2016 May 26;11(5):e0156076. doi: 10.1371/journal.pone.0156076 (PMC4881910; doi:10.1371/journal.pone.0156076)
Supplement: S3 Table — (PDF) [file pone.0156076.s004.pdf]

S3 Table: Risk factors associated with each questions.

Questions	Node Name(Network)	OR (95%CI)	*P*
Q1: How often did nurses treat you with courtesy and respect?	rx.metoprolol	5.61 (1.46, 21.51)	0.01
	div.bmt.oncology	3.76 (1.79, 7.89)	0.001
	dx.pneumonia	2.65 (1.3, 5.37)	0.007
	race.aa	1.68 (1.22, 2.31)	0.002
	lab.chloride.ahigh	1.44 (1.11, 1.87)	0.006
	cms.self.e.health	1.36 (1.23, 1.51)	<.0001
	cms.education	0.84 (0.78, 0.91)	<.0001
	rem.riskmodel	0.78 (0.7, 0.88)	<.0001
	dx.other.dis.stomach	0.34 (0.13, 0.91)	0.03
	lab.sodium.alowhigh	0.32 (0.15, 0.71)	0.005
	dx.viral.infection	0.24 (0.06, 0.94)	0.04
	race.asian	0.19 (0.1, 0.37)	<.0001
	rx.emtricitabine	0.17 (0.04, 0.73)	0.02
	rx.thiamine	0.14 (0.03, 0.65)	0.01
	rx.pregabalin	0.14 (0.04, 0.51)	0.003
	dx.hodgkin	0.06 (0.01, 0.82)	0.03
Q2: How often did nurses listen to you carefully?	rx.ergocalciferol	10.93 (1.3, 91.7)	0.03
	div.bmt.oncology	2.26 (1.25, 4.06)	0.007
	cms.self.e.health	1.3 (1.19, 1.42)	<.0001
	cms.education	0.85 (0.79, 0.91)	<.0001
	dx.infec.dz.2nd	0.79 (0.65, 0.97)	0.02
	cms.new.med	0.67 (0.54, 0.84)	0.0004
	admis.status.er	0.64 (0.48, 0.85)	0.002
	race.white	0.54 (0.42, 0.69)	<.0001
	cms.dis.facility	0.41 (0.26, 0.64)	<.0001
	rx.oxycodone	0.4 (0.22, 0.75)	0.004
	race.asian	0.31 (0.16, 0.59)	0.0004
	dx.other.dis.stomach	0.24 (0.08, 0.68)	0.008
	1proc.eye.or	0.07 (0.01, 0.63)	0.02

Q3: How often did nurses explain things in a way you could understand?	rx.polyethyleneclycol	5.12 (1.63, 16.12)	0.005
	lab.monocyte.alow	2.1 (1.15, 3.83)	0.02
	race.aa	1.78 (1.35, 2.35)	<.0001
	dx.ccs1.neoplasms	1.77 (1.22, 2.58)	0.003
	dis.status.home	1.71 (1.34, 2.17)	<.0001
	cms.puertorican	1.67 (1.17, 2.38)	0.005
	cms.self.e.health	1.29 (1.18, 1.42)	<.0001
	cms.education	0.89 (0.83, 0.95)	0.001
	admit.severity	0.85 (0.74, 0.97)	0.01
	rx.docusate	0.57 (0.33, 0.98)	0.04
	dx.anemia	0.48 (0.25, 0.94)	0.03
	race.asian	0.35 (0.19, 0.67)	0.001
	rx.pneumococcal.vac	0.34 (0.17, 0.69)	0.003
	rx.pregabalin	0.2 (0.05, 0.81)	0.02
Q4: How often did doctors treat you with courtesy and respect?	dx.pneumonia	2.77 (1.25, 6.16)	0.01
	cms.home	1.53 (1.07, 2.17)	0.02
	religion.catholic	1.36 (1.01, 1.84)	0.04
	cms.self.e.health	1.28 (1.15, 1.43)	<.0001
	age	1.01 (1, 1.02)	0.005
	cms.education	0.78 (0.72, 0.85)	<.0001
	lab.lymph.ahigh	0.61 (0.41, 0.92)	0.02
	admis.status.er	0.46 (0.33, 0.65)	<.0001
	rx.pneumococcal.vac	0.4 (0.19, 0.83)	0.01
	race.asian	0.32 (0.17, 0.62)	0.001
	dx.other.dis.stomach	0.26 (0.09, 0.73)	0.01
	dx.arthropathies	0.2 (0.05, 0.8)	0.02
	dx.chestpain	0.16 (0.05, 0.47)	0.001
	admis.status.na	0.1 (0.02, 0.59)	0.01
Q5: How often did doctors listen carefully to you?	lab.wbc.alowhigh	1.52 (1.01, 2.28)	0.04
	religion.catholic	1.37 (1.06, 1.78)	0.02
	cms.self.e.health	1.34 (1.22, 1.48)	<.0001
	cms.education	0.81 (0.75, 0.87)	<.0001
	cms.new.med	0.68 (0.53, 0.86)	0.001

	uhc.primary.hmo	0.64 (0.47, 0.87)	0.005
	admis.status.er	0.61 (0.45, 0.83)	0.001
	dx.ccs1.metabolic	0.56 (0.36, 0.87)	0.01
	lab.lymph.ahigh	0.63 (0.43, 0.91)	0.01
	cms.dis.facility	0.58 (0.37, 0.91)	0.02
	rx.pneumococcal.vac	0.39 (0.19, 0.8)	0.01
	rx.loratadine	0.33 (0.13, 0.79)	0.01
	rx.montelukast	0.28 (0.09, 0.85)	0.02
	rx.lorazepam	0.18 (0.06, 0.53)	0.002
	dx.other.dis.stomach	0.22 (0.08, 0.62)	0.004
	1proc.eye.or	0.04 (0, 0.31)	0.003
Q6: How often did doctors explain things in a way you could understand?	rx.esomeprazole	2.16 (1.04, 4.49)	0.04
	lab.sodium.alow	1.44 (1.13, 1.83)	0.003
	dis.day.weds	1.47 (1.1, 1.98)	0.01
	dis.status.home	1.51 (1.18, 1.93)	0.001
	cms.home	1.54 (1.13, 2.1)	0.007
	cms.self.e.health	1.34 (1.22, 1.48)	<.0001
	race.aa	1.32 (1, 1.74)	0.05
	cms.education	0.86 (0.81, 0.92)	<.0001
	admis.status.er	0.69 (0.53, 0.91)	0.009
	cms.new.med	0.69 (0.55, 0.87)	0.002
	lab.lymph.ahigh	0.66 (0.45, 0.95)	0.03
	uhc.2nd.commerial	0.61 (0.46, 0.81)	0.001
	dx.ccs1.metabolic	0.58 (0.38, 0.9)	0.02
	rx.pneumococcal.vac	0.43 (0.21, 0.88)	0.02
	rx.loratadine	0.38 (0.16, 0.88)	0.02
	race.asian	0.4 (0.21, 0.76)	0.005
	dx.other.dis.stomach	0.33 (0.12, 0.91)	0.03
	rx.trazodone	0.24 (0.09, 0.66)	0.005
Q7: How often were your room and bathroom kept clean?	lab.glu.alow	2.07 (1.29, 3.31)	0.003
	1proc.cardio.or	2.06 (1.14, 3.72)	0.02
	div.bmt.oncology	1.85 (1.16, 2.94)	0.009
	cms.self.e.health	1.24 (1.14, 1.35)	<.0001

	cms.education	0.83 (0.77, 0.88)	<.0001
	dx.ccs1.digestive	0.69 (0.53, 0.89)	0.005
	race.white	0.77 (0.61, 0.97)	0.03
	cms.new.med	0.74 (0.6, 0.91)	0.005
	dx.infec.dz.2nd	0.74 (0.61, 0.89)	0.002
	dx.intestinal.inf	0.43 (0.21, 0.86)	0.02
	race.asian	0.27 (0.14, 0.51)	<.0001
	rx.morphine	0.22 (0.08, 0.64)	0.005
	dx.other.endo.dis	0.15 (0.03, 0.78)	0.02
Q8: How often was the area around your room quiet at night?	rx.oxycodone	3.44 (1.38, 8.6)	0.008
	rx.furosemide	2.22 (1.27, 3.89)	0.005
	religion.catholic	1.43 (1.13, 1.82)	0.003
	div.bmt.oncology	2.35 (1.49-3.69)	0.0002
	race.aa	1.4 (1.04, 1.9)	0.03
	cms.self.e.health	1.2 (1.11, 1.31)	<.0001
	cms.education	0.84 (0.79, 0.9)	<.0001
	cms.new.med	0.7 (0.57, 0.86)	0.001
	race.white	0.6 (0.46, 0.79)	0.0003
	lab.k.alowhigh	0.49 (0.31, 0.78)	0.003
	race.asian	0.39 (0.19, 0.8)	0.01
	rx.multivit.min	0.12 (0.02, 0.66)	0.01
Q9: How often did you get help in getting to the bathroom or in using a bedpan as soon as you wanted?	cms.help.bath.bed	4.47 (2.9, 6.9)	<.0001
	cms.self.e.health	1.44 (1.25, 1.66)	<.0001
	cms.education	0.86 (0.78, 0.94)	0.002
	rem.riskmodel	0.85 (0.73, 0.99)	0.04
	lab.mcv.ahigh	0.67 (0.46, 0.98)	0.04
	religion.jewish	0.58 (0.36, 0.91)	0.02
	lab.chloride.n	0.59 (0.41, 0.85)	0.005
	admis.status.urgt	0.55 (0.32, 0.95)	0.03
	race.asian	0.35 (0.12, 1)	0.05
	rx.enoxaparin	0.09 (0.01, 0.71)	0.02
Q10: After you pressed the call bell, how often did you get help as soon as you wanted?	dx.abdomian.pain	9.33 (1.15, 75.88)	0.04
	div.bmt.oncology	2.86 (1.79, 4.57)	<.0001

	cms.puertorican	2.21 (1.52, 3.2)	<.0001
	lab.wbc.n	1.36 (1.05, 1.76)	0.02
	dis.status.home	1.34 (1.03, 1.75)	0.03
	cms.self.e.health	1.26 (1.14, 1.39)	<.0001
	num.cc	0.97 (0.95, 0.99)	0.001
	rem.riskmodel	0.89 (0.79, 0.99)	0.05
	dx.mental.dis.2nd	0.82 (0.7, 0.95)	0.008
	cms.education	0.82 (0.76, 0.88)	<.0001
	dx.infec.dz.2nd	0.69 (0.55, 0.86)	0.001
	dx.other.gi	0.33 (0.12, 0.86)	0.02
Q11: How often was your pain well controlled?	dx.diabetes	2.65 (1.03, 6.82)	0.04
	dx.ccs1.neoplasms	2.39 (1.58, 3.62)	<.0001
	cms.home	1.69 (1.17, 2.44)	0.006
	religion.catholic	1.66 (1.25, 2.2)	0.001
	lab.eos.alow	1.53 (1.17, 2)	0.002
	cms.self.e.health	1.19 (1.06, 1.32)	0.002
	rem.riskmodel	0.86 (0.75, 0.98)	0.03
	uhc.primary.medicare	0.7 (0.53, 0.92)	0.01
	cms.new.med	0.65 (0.49, 0.86)	0.003
	race.white	0.62 (0.47, 0.81)	0.001
	lab.monocyte.alow	0.45 (0.24, 0.83)	0.010
	dx.syncope	0.38 (0.16, 0.91)	0.03
	rx.zolpidem	0.29 (0.1, 0.83)	0.02
	rx.pregabalin	0.16 (0.03, 0.79)	0.02
	race.asian	0.14 (0.05, 0.41)	0.0003
Q12: How often did the hospital staff do everything they could to help you with your pain?	1proc.lymphatic.or	4.65 (2.02, 10.72)	0.0003
	lab.hg.ahigh	4.59 (1.34, 15.67)	0.02
	dx.diabetes	3.07 (1.06, 8.9)	0.04
	cms.self.e.health	1.19 (1.06, 1.34)	0.003
	cms.nonhispanic	0.72 (0.52, 0.99)	0.05
	lab.cr.ahigh	0.62 (0.44, 0.88)	0.007
	cms.dis.facility	0.59 (0.35, 1)	0.05
	race.white	0.57 (0.41, 0.79)	0.001

	rx.oxycodone	0.43 (0.21, 0.86)	0.02
	race.asian	0.26 (0.11, 0.62)	0.002
	dx.other.dis.stomach	0.2 (0.05, 0.84)	0.03
	rx.pregabalin	0.18 (0.05, 0.68)	0.01
Q13: Before giving you the meds, how often did hospital staff tell you what the medicine was for?	div.bmt.oncology	2.3 (1.12, 4.72)	0.02
	dx.ccs1.neoplasms	2.08 (1.27, 3.41)	0.004
	dis.status.home	1.92 (1.44, 2.55)	<.0001
	cms.self.e.health	1.23 (1.09, 1.39)	0.001
	cms.education	0.86 (0.79, 0.95)	0.001
	dx.neoplasm.2nd	0.72 (0.56, 0.93)	0.01
	cms.nonhispanic	0.73 (0.53, 1)	0.05
	dx.infec.dz.2nd	0.77 (0.61, 0.97)	0.03
	lab.cr.ahigh	0.68 (0.5, 0.94)	0.02
	lab.baso.alowhigh	0.52 (0.36, 0.76)	0.001
	race.asian	0.34 (0.16, 0.71)	0.004
	dx.cong.hf	0.33 (0.12, 0.9)	0.03
	dx.viral.infection	0.1 (0.02, 0.57)	0.009
	rx.vancomycin	0.2 (0.05, 0.88)	0.03
Q14: Before giving you the new meds, how often did hospital staff describe side effects in a way you could understand?	rx.warfarin	3.53 (1.36, 9.2)	0.010
	lab.lymph.n	2.06 (1.27, 3.35)	0.004
	cms.self.e.health	1.22 (1.1, 1.36)	0.0002
	cms.education	0.85 (0.78, 0.92)	<.0001
	race.white	0.69 (0.52, 0.92)	0.01
	uhc.2nd.commerial	0.62 (0.43, 0.89)	0.010
	admis.status.er	0.51 (0.38, 0.69)	<.0001
Q15: Did hospital staff talk with you about whether you would have the help you needed when you left the hospital?	lab.calcium.ahigh	5.11 (1.19, 21.94)	0.03
	admit.day.tue	1.77 (1.21, 2.58)	0.003
	cms.help.bath.bed	1.61 (1.17, 2.21)	0.003
	lab.neut.alowhigh	1.58 (1.02, 2.43)	0.04
	los.obs	1.04 (1.01, 1.07)	0.009
	cms.education	0.89 (0.82, 0.97)	0.005
	gender.female	0.69 (0.53, 0.9)	0.007
	admis.status.er	0.6 (0.42, 0.85)	0.005

	dis.day.sun	0.58 (0.37, 0.9)	0.02
	religion.jewish	0.55 (0.39, 0.78)	0.001
	dis.status.home	0.43 (0.3, 0.62)	<.0001
	dx.intesi.obs	0.23 (0.06, 0.87)	0.03
Q16: Did you get information in writing about what symptoms/health problems to look out for after you left the hospital?	admit.severity	2.11 (1.52, 2.94)	<.0001
	arm	1.45 (1.03, 2.05)	0.03
	los.obs	1.1 (1.04, 1.16)	0.001
	los.exp	1.07 (1, 1.15)	0.05
	num.dx	1.05 (1.01, 1.09)	0.03
	lab.calcium.n	0.53 (0.32, 0.86)	0.01
	num.cc	0.43 (0.39, 0.48)	<.0001
	dx.uc	0.31 (0.15, 0.65)	0.002
	rx.acetaminophen	0.14 (0.04, 0.49)	0.002
Q17: What number would you use to rate this hospital? (0: worst;10: best)	nycounty.kins	1.49 (1.04, 2.14)	0.03
	cms.self.e.health	1.36 (1.25, 1.49)	<.0001
	cms.education	0.77 (0.72, 0.82)	<.0001
	cms.new.med	0.73 (0.59, 0.9)	0.003
	admis.status.er	0.61 (0.47, 0.78)	<.0001
	uhc.primary.hmo	0.58 (0.42, 0.79)	0.001
	race.white	0.57 (0.45, 0.72)	<.0001
	cms.dis.facility	0.48 (0.3, 0.78)	0.003
	1proc.cardio.or	0.44 (0.25, 0.77)	0.004
	lab.mch.alow	0.39 (0.17, 0.93)	0.03
	dx.urinary.t.inf	0.19 (0.05, 0.67)	0.01
Q18: Would you recommend this hospital to your friends and family?	div.bmt.oncology	2.48 (1.38, 4.44)	0.002
	lab.sodium.alow	1.56 (1.23, 1.97)	0.0002
	cms.home	1.52 (1.11, 2.07)	0.009
	dis.status.home	1.41 (1.11, 1.81)	0.006
	religion.catholic	1.3 (1.02, 1.67)	0.04
	cms.self.e.health	1.22 (1.11, 1.34)	<.0001
	cms.education	0.89 (0.83, 0.96)	0.002
	race.white	0.62 (0.48, 0.79)	0.0001
	rx.oxycodone	0.29 (0.16, 0.54)	<.0001

	race.asian	0.28 (0.15, 0.54)	0.0002
	rx.emtricitabine	0.19 (0.05, 0.83)	0.03
