# Supplementary material for: Data-Driven Identification of Risk Factors of Patient Satisfaction at a Large Urban Academic Medical Center
Source: PLoS One. 2016 May 26;11(5):e0156076. doi: 10.1371/journal.pone.0156076 (PMC4881910; doi:10.1371/journal.pone.0156076)
Supplement: S4 Table — (DOCX) [file pone.0156076.s005.docx]

**S4 Table: Data Dictionary used in Network and Tables**

| **Node Name(Network)** | **Description** |
| --- | --- |
| 1proc.cardio.or | first procedure category_Operations on the cardiovascular system |
| 1proc.eye.or | first procedure category_Operations on eye |
| 1proc.lymphatic.or | first procedure category_Operations on the hemic and lymphatic system |
| admis.status.er | admission status_ER |
| admis.status.na | admission status_n/a |
| admis.status.urgt | admission status_Urgent |
| admit.day.tue | admit day _Tuesday |
| admit.severity | Admit Severity of Illness |
| age | age |
| arm | Admit Risk of Mortality |
| cms.dis.facility | cms_discharge to another facility |
| cms.education | cms_education_level |
| cms.help.bath.bed | cms_help to bathroom or bed |
| cms.home | cms_discharge to home |
| cms.new.med | cms_on new medication |
| cms.nonhispanic | cms_not hispanic |
| cms.puertorican | cms_origin or descent:puerto rican |
| cms.self.e.health | cms_self_evaluation.health |
| dis.day.sun | discharged day_Sunday |
| dis.day.weds | discharged day_Wednesday |
| dis.status.home | discharge status_home |
| div.bmt.oncology | division_bmt_oncology |
| dx.mental.dis.2nd | secondary diagnosis_mental disorders |
| dx.abdomian.pain | diagnosis_abdominal pain |
| dx.anemia | diagnosis_deficiency and other anemia |
| dx.arthropathies | diagnosis_gout and other crystal arthropathies |
| dx.ccs1.digestive | diagnosis_disease category level 1(ccs1)_digestive disease |
| dx.ccs1.metabolic | diagnosis_disease category level 1(ccs1)_metabolic disease |
| dx.ccs1.neoplasms | diagnosis_neoplasms |
| dx.chestpain | diagnosis_nonspecific chest pain |
| dx.cong.hf | diagnosis_congestive heart failure; nonhypertensive |
| dx.diabetes | diagnosis_diabetes mellitus with complications |
| dx.hodgkin | diagnosis_hodgkin's disease |
| dx.infec.dz.2nd | secondary diagnosis_Infectious and parasitic diseases |
| dx.intesi.obs | diagnosis_intestinal obstruction without hernia |
| dx.intestinal.inf | diagnosis_intestinal infection |
| dx.neoplasm.2nd | secondary diagnosis_neoplasm |
| dx.other.dis.stomach | diagnosis_other disorders of stomach and duodenum |
| dx.other.endo.dis | diagnosis_other endocrine disorders |
| dx.other.gi | diagnosis_other gastrointestinal disorders |
| dx.pneumonia | diagnosis_pneumonia(not by TB and sexually) |
| dx.syncope | diagnosis_syncope |
| dx.uc | diagnosis_regional enteritis and ulcerative colitis |
| dx.urinary.t.inf | diagnosis_urinary tract infections |
| dx.viral.infection | diagnosis_viral infection |
| gender.female | gender_female |
| lab.baso.alowhigh | labs_basophil %_abnormal_low_high |
| lab.calcium.ahigh | labs_calcium_abnormal_high |
| lab.calcium.n | labs_calcium_normal |
| lab.chloride.ahigh | labs_chloride_abnormal_high |
| lab.chloride.n | labs_chloride_normal |
| lab.cr.ahigh | labs_creatinine_abnormal_high |
| lab.eos.alow | labs_eosinophil %_abnormal_low |
| lab.glu.alow | labs_glucose_abnormal_low |
| lab.hg.ahigh | labs_hemoglobin_abnormal_high |
| lab.k.alowhigh | labs_potassium_abnormal_low_high |
| lab.lymph.ahigh | labs_lymphocyte %_abnormal_high |
| lab.lymph.n | labs_lymphocyte %_normal |
| lab.mch.alow | labs_mean_corp_hbg_abnormal_low |
| lab.mcv.ahigh | labs_mean_corp_volume_abnormal_high |
| lab.monocyte.alow | labs_monocyte counts_abnormal_low |
| lab.neut.alowhigh | labs_neutrophil %_abnormal_low_high |
| lab.sodium.alow | labs_sodium_abnormal_low |
| lab.sodium.alowhigh | labs_sodium_abnormal_low_high |
| lab.wbc.alowhigh | labs_wbc_abnormal_low_high |
| lab.wbc.n | labs_wbc_normal |
| los.exp | length_of_stay_expected (2012 risk model) |
| los.obs | length_of_stay_observed |
| num.cc | number of comorbidity and complications |
| num.dx | # of diagnosis |
| nycounty.kins | New York County_Kings |
| race.aa | race_african-american |
| race.asian | race_asian |
| race.white | race_white |
| religion.catholic | religion_catholic |
| religion.jewish | religion_jewish |
| rem.riskmodel | Relative Expected Mortality (2012 Risk Model) |
| rx.acetaminophen | medication_acetaminophen |
| rx.docusate | medication_docusate sodium |
| rx.emtricitabine | medication_emtricitabine/tenofovir |
| rx.enoxaparin | medication_enoxaparin sodium |
| rx.ergocalciferol | medication_ergocalciferol(vitamin D2) |
| rx.esomeprazole | medication_esomeprazole capsule |
| rx.furosemide | medicaion_furosemide |
| rx.loratadine | medication_loratadine |
| rx.lorazepam | medication_lorazepam injection |
| rx.metoprolol | medication_metoprolol |
| rx.montelukast | medication_montelukast sodium |
| rx.morphine | medication_morphine injection |
| rx.multivit.min | medication_multivitamin_Tx-Iron-Ca-FA-Min_Oral |
| rx.oxycodone | medication_oxycodone/acetaminophen |
| rx.pneumococcal.vac | medicaion_pneumococcal 23_val p_sac vac |
| rx.polyethyleneclycol | medication_polyethylene glycol |
| rx.pregabalin | medication_pregabalin |
| rx.thiamine | medication_thiamine |
| rx.trazodone | medication_trazodone_hcl |
| rx.vancomycin | medication_vancomycin injection |
| rx.warfarin | medication_warfarin sodium |
| rx.zolpidem | medication_zolpidem |
| uhc.2nd.commerial | UHC secondary payer_commerial |
| uhc.primary.hmo | UHC primary payer_HMO |
| uhc.primary.medicare | UHC primary_payer_medicare traditional_indemnity |
